# Supplementary material for: Small extracellular vesicles in follicular fluids for predicting reproductive outcomes in assisted reproductive technology
Source: Commun Med (Lond). 2024 Feb 28;4:33. doi: 10.1038/s43856-024-00460-8 (PMC10902298; doi:10.1038/s43856-024-00460-8)
Supplement: Supplementary file 2 — Supplementary information [file 43856_2024_460_MOESM2_ESM.pdf]

Title: **Small extracellular vesicles in follicular fluids for predicting reproductive outcomes in assisted reproductive technology**

Ayako Muraoka, Akira Yokoi*, Kosuke Yoshida, Masami Kitagawa, Eri Asano-Inami, Mayuko Murakami, Bayasula Bayasula, Natsuki Miyake, Natsuki Nakanishi, Tomoko Nakamura, Satoko Osuka, Akira Iwase, and Hiroaki Kajiyama

Supplementary information

List of Supplementary Materials

Supplementary Figure S1 to S5

Legend of Supplementary Figure S1 to S5

Supplementary Data S1

Supplementary Data S2

Supplementary Table S1

a

Patient No.	FF sample No.	pregnant/non-pregnant	Veeck clasification	Gardner classification
Patient 4	P1	pregnant	G1	
Patient 1	P2	pregnant	G3	
Patient 5	P3	pregnant	G2	
Patient 2	P4	pregnant	G1	
Patient 2	P5	pregnant	G3	
Patient 6	P6	pregnant	G1	
Patient9	P9	pregnant	G1	
Patient 3	P10	pregnant		3CB
Patient10	N1	non-pregnant	G1	
Patient11	N2	non-pregnant	G1	
Patient12	N3	non-pregnant	G1	
Patient13	N4	non-pregnant	G1	
Patient14	N5	non-pregnant		4AA
Patient17	N8	non-pregnant	G1	
Patient18	N9	non-pregnant	G1	
Patient 1	N10	non-pregnant	G3	
Patient 1	N11	non-pregnant	G3	
Patient 2	N12	non-pregnant	G3	
Patient 3	N13	non-pregnant		4CC
Patient19	N14	non-pregnant		4CB

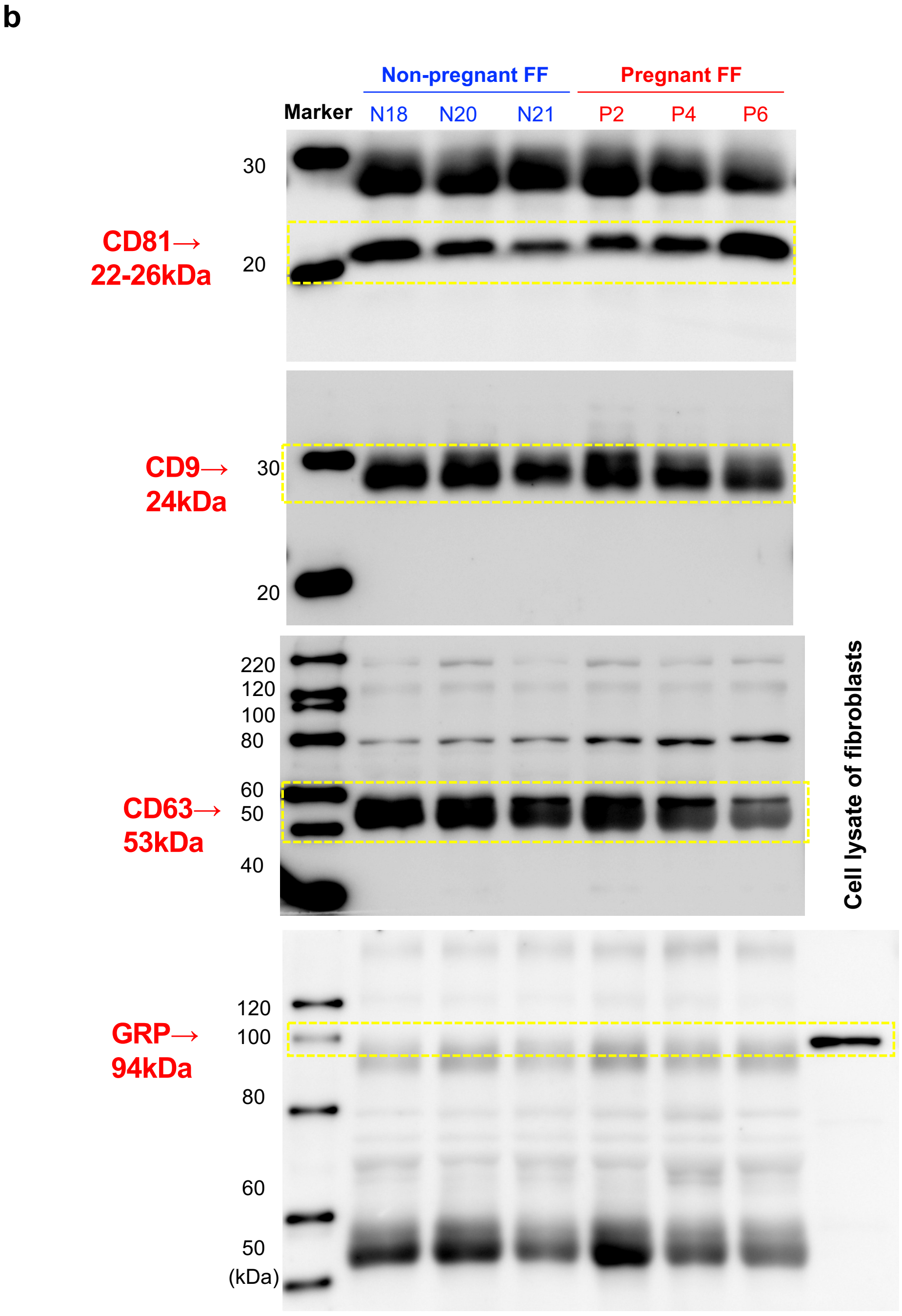
b
Non-pregnant FF
Pregnant FF
Marker
N18
N20
N21
P2
P4
P6
CD81→
22-26kDa
30
20
CD9→
24kDa
30
20
220
120
100
80
60
50
40
CD63→
53kDa
Cell lysate of fibroblasts
GRP→
94kDa
120
100
80
60
50
(kDa)

a

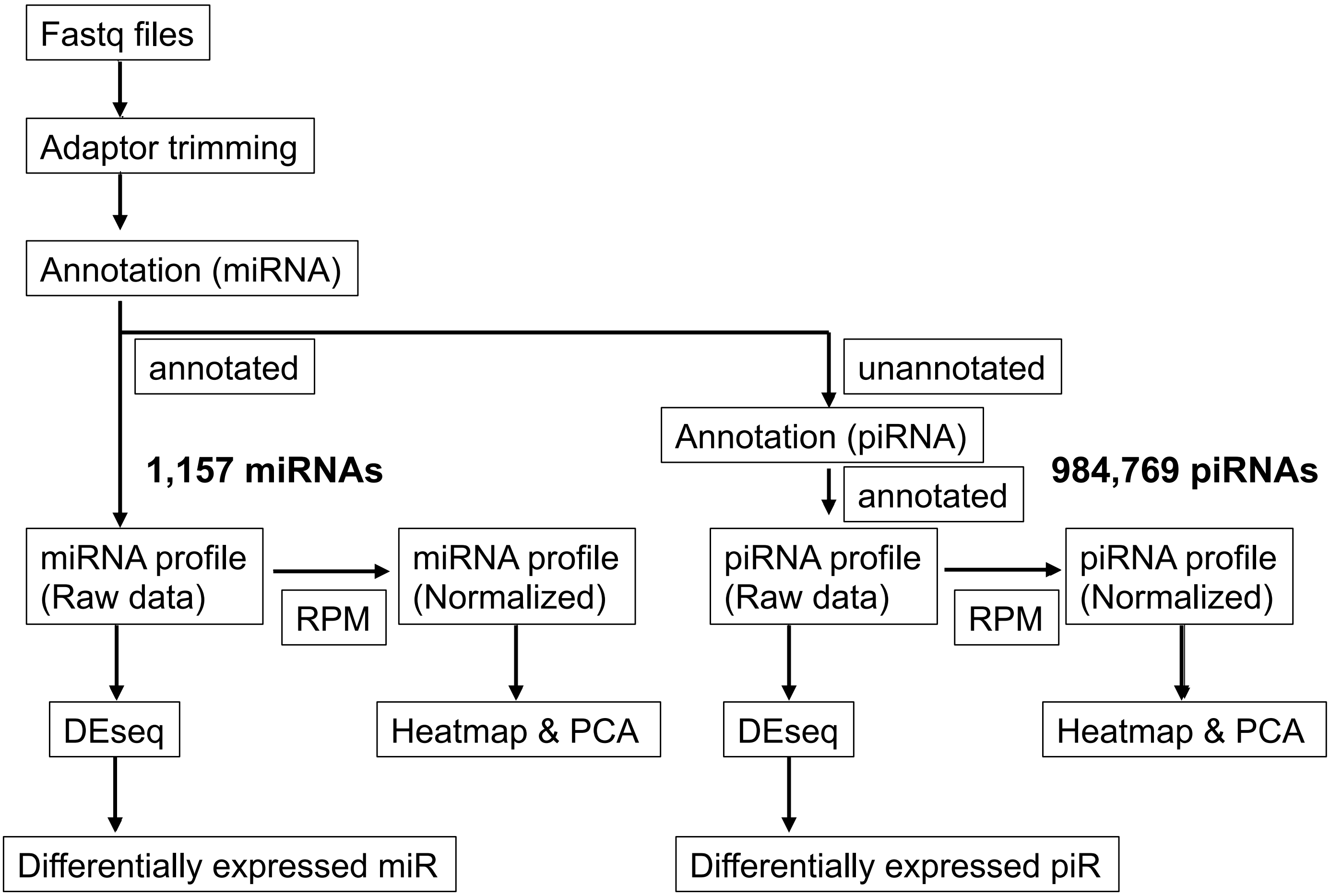
Fastq files
Adaptor trimming
Annotation (miRNA)
annotated
unannotated
Annotation (piRNA)
annotated
1,157 miRNAs
984,769 piRNAs
miRNA profile (Raw data)
RPM
miRNA profile (Normalized)
piRNA profile (Raw data)
RPM
piRNA profile (Normalized)
DEseq
Heatmap & PCA
DEseq
Heatmap & PCA
Differentially expressed miR
Differentially expressed piR

Supplementary Figure S4

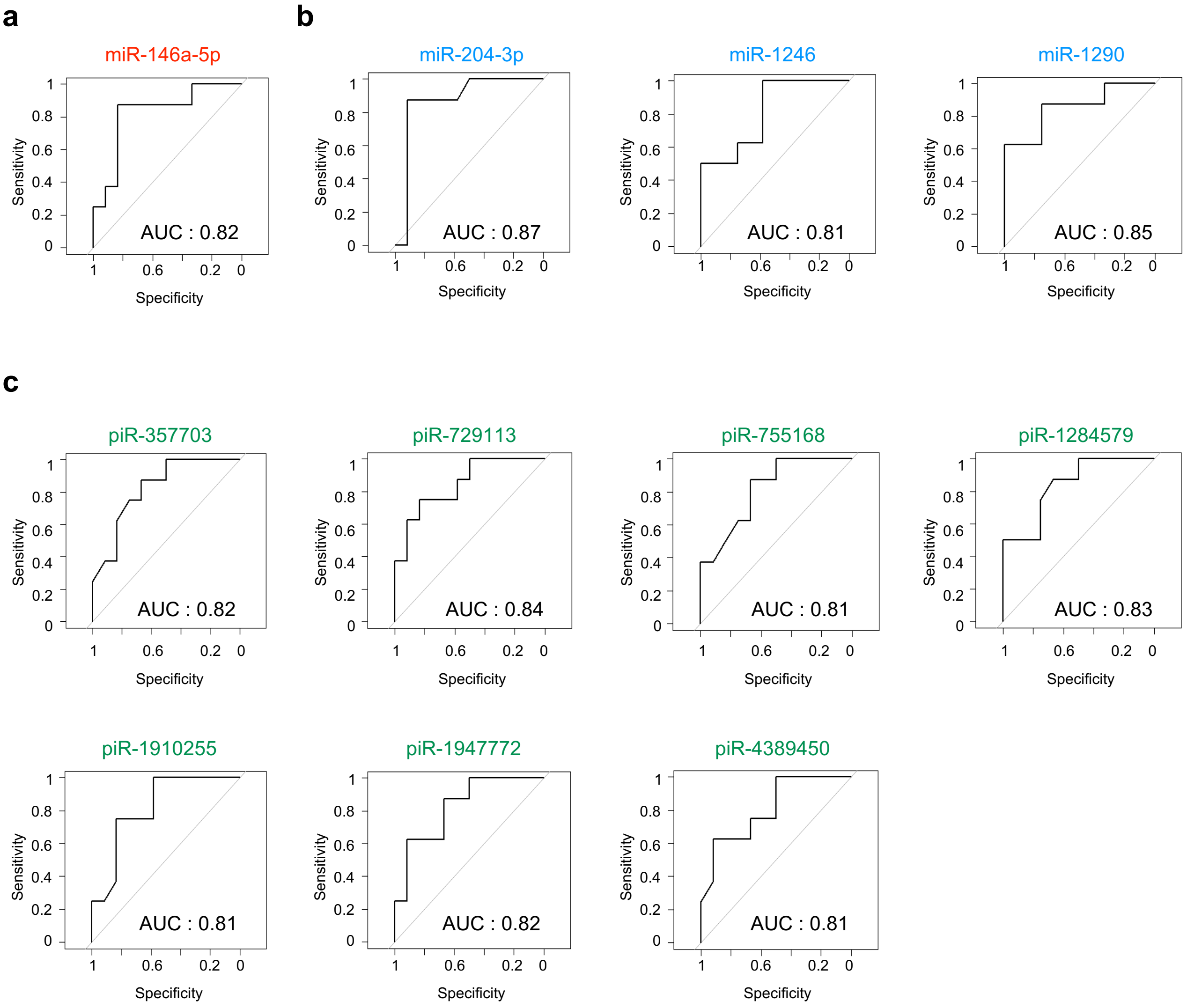

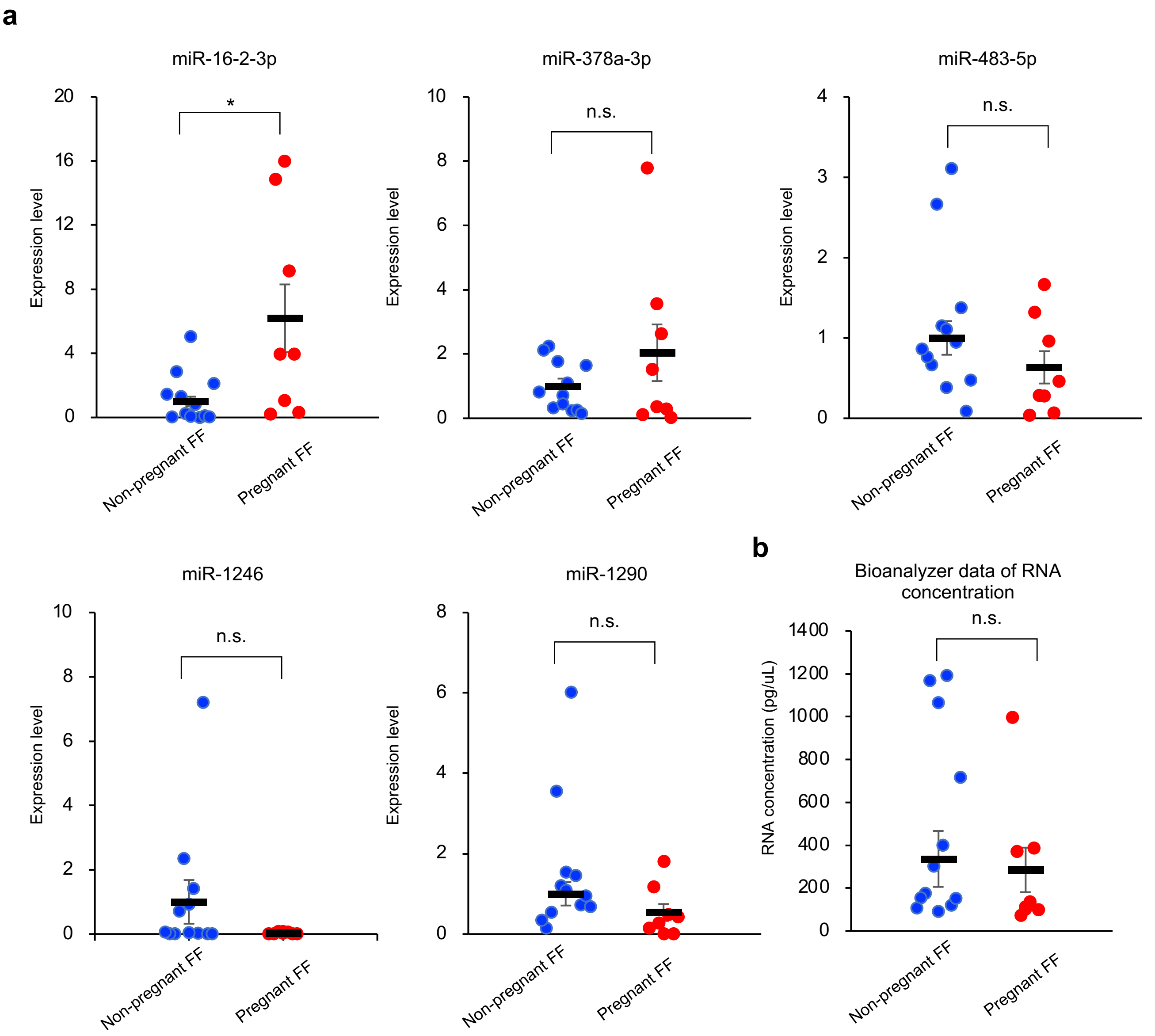
a
miR-16-2-3p
Expression level
*
Non-pregnant FF
Pregnant FF
miR-378a-3p
n.s.
miR-483-5p
n.s.
miR-1246
n.s.
miR-1290
n.s.
b
Bioanalyzer data of RNA concentration
RNA concentration (pg/uL)
n.s.

Legend of Supplementary Figure S1 to S5

Supplementary Fig. S1. Correspondence table for patient, FF samples, pregnancy status, and embryo classification.

(**a**) Table with patient number, FF samples number, pregnancy status, and embryo classification, respectively.

Supplementary Fig. S2. Uncropped blots

(**a**) Uncropped blots for western blotting. Cell lysate of fibroblasts was used as the positive control for GRP.

Supplementary Fig. S3. Flowchart of the process of small RNA sequencing

(**a**) Flowchart of analysis method in this study.

Supplementary Fig. S4. ROC curves for small ncRNAs

(**a**) Upregulated and (**b**) downregulated miRNAs in the pregnant group. (**c**) Downregulated piRNAs in the pregnant group.

Supplementary Fig. S5. qPCR validation and RNA concentration in FF-sEVs

(**a**) miRNA expression in pregnant group (n = 8) and non-pregnant group (n = 12). The y-axis indicates the miRNA expression normalized by RNA content. (**b**) RNA concentration in pregnant group (n = 8) and non-pregnant group (n = 12) measured by Bioanalyzer. *, $P < 0.05$., n.s., not significant. The data were analyzed by the student's *t*-test.

Supplementary Data S1. Upregulated and downregulated piRNAs in pregnancy group

(in Excel file)

Supplementary Data S2. The source data for the figures

(in Excel file)

Supplementary Table S1. Upregulated and downregulated miRNAs in pregnancy group.

miRNA	Log2FC	p-value	Average read count			AUC
			Pregnancy group	Non-pregnancy group	All samples	
**<Upregulated>**						
hsa-miR-16-2-3p	0.833	0.0026	1800	955	1293	0.854
hsa-miR-146a-5p	1.070	0.0058	1838	814	1224	0.823
hsa-miR-11400	3.988	0.0025	60	3	26	0.797
**<Downregulated>**						
hsa-miR-27a-5p	-2.003	0.0012	20	70	50	0.786
hsa-miR-203a-3p	-1.760	0.0031	2434	7392	5408	0.688
hsa-miR-203b-5p	-1.886	0.0025	2259	7474	5388	0.719
hsa-miR-204-3p	-1.812	0.0002	65	208	151	0.870
hsa-miR-378a-3p	-1.018	0.0012	954	1642	1367	0.901
hsa-miR-455-3p	-5.197	0.0034	0	13	8	0.328
hsa-miR-483-5p	-1.475	0.0012	4991	11790	9070	0.854
hsa-miR-514a-3p	-1.072	0.0021	5753	10204	8424	0.771
hsa-miR-1246	-1.025	0.0012	1581	2715	2261	0.813
hsa-miR-1290	-1.280	0.0002	593	1196	955	0.854

FC, fold change; AUC, area under the curve.

Log2FC and p-value were calculated using raw read counts with the DEseq2 package of R software.
Average read count and AUC were calculated using normalized read counts.
