# Supplementary material for: Small extracellular vesicles in follicular fluids for predicting reproductive outcomes in assisted reproductive technology
Source: Commun Med (Lond). 2024 Feb 28;4:33. doi: 10.1038/s43856-024-00460-8 (PMC10902298; doi:10.1038/s43856-024-00460-8)
Supplement: Supplementary file 3 — Description of Additional Supplementary Files [file 43856_2024_460_MOESM3_ESM.pdf]

Description of Additional Supplementary Files

File Name: Supplementary Data S1
Description: Upregulated and downregulated piRNAs in pregnancy group

File Name: Supplementary Data S2
Description: The source data for the figures
